# Association Between Furosemide Exposure and Clinical Outcomes in a Retrospective Cohort of Critically Ill Children

**DOI:** 10.3389/fped.2020.589124

**Published:** 2021-01-25

**Authors:** Xiaomei Dai, Jiao Chen, Wenjing Li, Zhenjiang Bai, Xiaozhong Li, Jian Wang, Yanhong Li

**Affiliations:** ^1^Department of Nephrology and Immunology, Children's Hospital of Soochow University, Suzhou, China; ^2^Pediatric Intensive Care Unit, Children's Hospital of Soochow University, Suzhou, China; ^3^Institute of Pediatric Research, Children's Hospital of Soochow University, Suzhou, China

**Keywords:** mortality, furosemide, fluid overload, critically ill children, acute kidney injury

## Abstract

Furosemide is commonly prescribed in critically ill patients to increase the urine output and prevent fluid overload (FO) and acute kidney injury (AKI), but not supported by conclusive evidence. There remain conflicting findings on whether furosemide associates with AKI and adverse outcomes. Information on the impact of furosemide on adverse outcomes in a general population of pediatric intensive care unit (PICU) is limited. The aim of the cohort study was to investigate the associations of furosemide with AKI and clinical outcomes in critically ill children.

**Study Design:** We retrospectively reviewed a cohort of 456 critically ill children consecutively admitted to PICU from January to December 2016. The exposure of interest was the use of furosemide in the first week after admission. FO was defined as ≥5% of daily fluid accumulation, and mean FO was considered significant when mean daily fluid accumulation during the first week was ≥5%. The primary outcomes were AKI in the first week after admission and mortality during PICU stay. AKI diagnosis was based on Kidney Disease: Improving Global Outcomes criteria with both serum creatinine and urine output.

**Results:** Furosemide exposure occurred in 43.4% of all patients (*n* = 456) and 49.3% of those who developed FO (*n* = 150) in the first week after admission. Patients who were exposed to furosemide had significantly less degree of mean daily fluid accumulation than those who were not (1.10 [−0.33 to 2.61%] vs. 2.00 [0.54–3.70%], *P* < 0.001). There was no difference in the occurrence of AKI between patients who did and did not receive furosemide (22 of 198 [11.1%] vs. 36 of 258 [14.0%], *P* = 0.397). The mortality rate was 15.4% (70 of 456), and death occurred more frequently among patients who received furosemide than among those who did not (21.7 vs. 10.5%, *P* = 0.002). Furosemide exposure was associated with increased odds for mortality in a multivariate logistic regression model adjusted for body weight, gender, illness severity assessed by PRISM III score, the presence of mean FO, and AKI stage [adjusted odds ratio (AOR) 1.95; 95%CI, 1.08–3.52; *P* = 0.026].

**Conclusion:** Exposure to furosemide might be associated with increased risk for mortality, but not AKI, in critically ill children.

## Introduction

Acute kidney injury (AKI) is a common clinical syndrome and associated with high risk of morbidity and mortality in critically ill patients (1, 2). Children in the pediatric intensive care unit (PICU) experience an increased incidence of AKI (3–5), and critically ill children with AKI experience a higher mortality rate than those with AKI out of PICU (3). The common causes of AKI in critically ill children are secondary. Sepsis, multiorgan failure, shock, hemato-oncological diseases, post-cardiac surgery, and exposure to nephrotoxic medication, and mechanical ventilation (MV) are the main causes of AKI in the critically ill child (3, 6). Given the complexity of the disease in pathogenesis and the individual difference in the children in PICU, excessive intravenous fluids might worsen the development of fluid overload (FO) and AKI (7), even though the early fluid resuscitation is one of the fundamental measures of rescue therapy (8). Additionally, FO has been proven to be associated with AKI and predictive of hospital mortality in pediatrics (3, 9–11). Early recognition and treatment of FO and AKI are becoming a major clinical focus among critically ill children.

Loop diuretics, such as furosemide in particular, are commonly used for the treatment of edema of hepatic, renal, or cardiac origin (12–14). Although the exact mechanism of action is not fully understood, furosemide is believed to act on the lumen surface of the ascending wing of Henley loop by inhibiting the active reabsorption of chloride ions (13). In clinical practice, furosemide is often used in patients with or without AKI in the intensive care unit (ICU) in an attempt to increase urine output and prevent FO and AKI. However, the findings from the studies regarding the relationship between furosemide and fluid balance, AKI, and mortality in critically ill patients are inconclusive and controversial (15–23), which remains inadequately described in pediatrics. In particular, the data on whether furosemide can effectively reduce FO, and therefore reducing the occurrence of AKI and improving clinical outcomes in critically ill children, are scarce. The purpose of the study was to investigate the associations of furosemide with FO, AKI, and mortality in critically ill children, in order to assess whether critically ill children exposure to furosemide are less likely to experience AKI and mortality during the PICU stay.

## Patients and Methods

### Cohorts, Setting, and Data Collection

We conducted a retrospective review of all patients admitted to the PICU in Children's Hospital of Soochow University from January to December 2016. All patients met the criteria for PICU admission as described previously (24). The exclusion criteria were age of <28 days or more than 16 years; PICU length of stay of <24 h, including patients who did not survive, withdrew therapy, or were transferred to another hospital; incomplete clinical data on calculating FO; undergoing surgery due to congenital heart diseases; and receiving furosemide but after AKI. In addition, for patients with multiple PICU admissions within a single hospital stay, only the last admission, which was associated with the prognosis, was included in the analysis. The Ethics Committees of the Children's Hospital of Soochow University approved the study protocol, and the study was performed in accordance with the Declaration of Helsinki.

Demographic, laboratory, and clinical data were collected from the electronic medical record and included age, body weight, gender, and admission diagnosis. Clinical status, MV, renal replacement therapy (RRT), medication exposures, and length of stay were recorded until hospital discharge or death. Vasopressor exposure was defined as the use of any vasoactive agent, including dopamine, dobutamine, epinephrine, norepinephrine, vasopressin, milrinone, digoxin, and cedilanid during the PICU stay.

### Furosemide Administration

Information on the use of furosemide during the first week after PICU admission was recorded, and the cumulative doses per kilogram of body weight and frequencies in the initial week were calculated. The intermittent injection of furosemide was prescribed at the discretion of the attending physicians in the fields of critical care medicine to promote diuresis and prevent and reduce FO.

### Assessment of Illness Severity

Illness severity of critically ill children was assessed using the pediatric risk of mortality III (PRISM III) score, which was calculated based on the most abnormal values of physiological parameters collected in the first 24 h after PICU admission (25).

### Assessment of Fluid Overload

The presence and severity of FO were evaluated during the first week after PICU admission. FO was defined as ≥5% of daily fluid accumulation (10, 26). The calculation formula is as follows: Fluid accumulation (%) = [liquid input (L) – liquid output (L)]/body weight at PICU admission (kg) × 100% (27, 28). Taking the time of patients entering the PICU as the node, we recorded the daily fluid accumulation every 24 h and evaluated for the first week continuously. A maximum daily fluid accumulation ≥5% occurring during the first week after PICU admission was considered to be maximum FO. Mean daily fluid accumulation during the first week after admission was calculated, and mean FO was considered significant when mean daily fluid accumulation was ≥5%. Liquid input included intravenous rehydration fluid, nutrient fluid (intravenous or intestinal), blood products, oral drugs, etc. Liquid output included urine volume, drainage fluid, ultrafiltration volume during RRT, fecal volume, and estimated bleeding loss, etc. Invisible dehydration through the skin and lungs is excluded.

### Diagnosis of Acute Kidney Injury

AKI that developed during the first week after admission was diagnosed and classified according to the Kidney Disease: Improving Global Outcomes (KDIGO) clinical practice guidelines with both serum creatinine (SCr) and urine output (5, 29). When the two criteria of SCr and urine output resulted in different KDIGO stages, the higher stage was chosen. Baseline SCr was defined as the lowest SCr level within 6 months prior to PICU admission. If it was not available, the baseline SCr was defined as the lowest among SCr values in hospital but prior to PICU admission, the first SCr measurement on PICU admission, or the lowest SCr value within 2 weeks in the PICU for patients who presented to the PICU with an elevated SCr >106.1 μmol/L, successively, in accordance with our previous studies (9, 10). Notably, we are interested in furosemide exposure before AKI. If furosemide was used in the first week of admission but after AKI occurred, then those patients were excluded.

### Clinical Outcomes

The primary outcomes were AKI developed during the first week after admission and PICU mortality defined as all-cause mortality occurring during the PICU stay, including death resulting from withdrawal of therapy. Secondary outcomes were the length of stay of PICU and hospital.

### Statistical Analysis

We checked the assumptions of normality and homogeneity of variance first. All continuous variables were skewed distributed, described as the median and interquartile range (IQR), and compared using the Mann–Whitney *U* or the Kruskal–Wallis *H*-test. Categorical variables, described as counts and percentages, were compared using the chi-square or Fisher's exact test. Spearman's analysis was used to examine correlations. Univariate and stepwise multivariate linear regression analyses were performed to investigate the association of furosemide exposure with mean FO and secondary clinical outcomes. Continuous variables were log-transformed to meet the requirements of normal distribution. Collinearity diagnostics were used to evaluate whether there exists multicollinearity among variables. Variance inflation factor (VIF) ≥2 and tolerance ≤ 0.5 indicate the presence of significant multicollinearity in the multivariate linear regression model. Univariate and multivariate logistic regression models were performed to calculate the odds ratio (OR) and adjusted OR (AOR) with a 95% confidence interval (CI) to assess the association between furosemide exposure and mortality. Body weight, gender, PRISM III score, the presence of mean FO, and AKI stage were entered into the multivariate logistic regression model for covariate adjustment. The two-sided significance level was set at 0.05. All data analyses were performed using the statistical software of SPSS 21.0.

## Results

### Patient Characteristics

**Figure 1:**
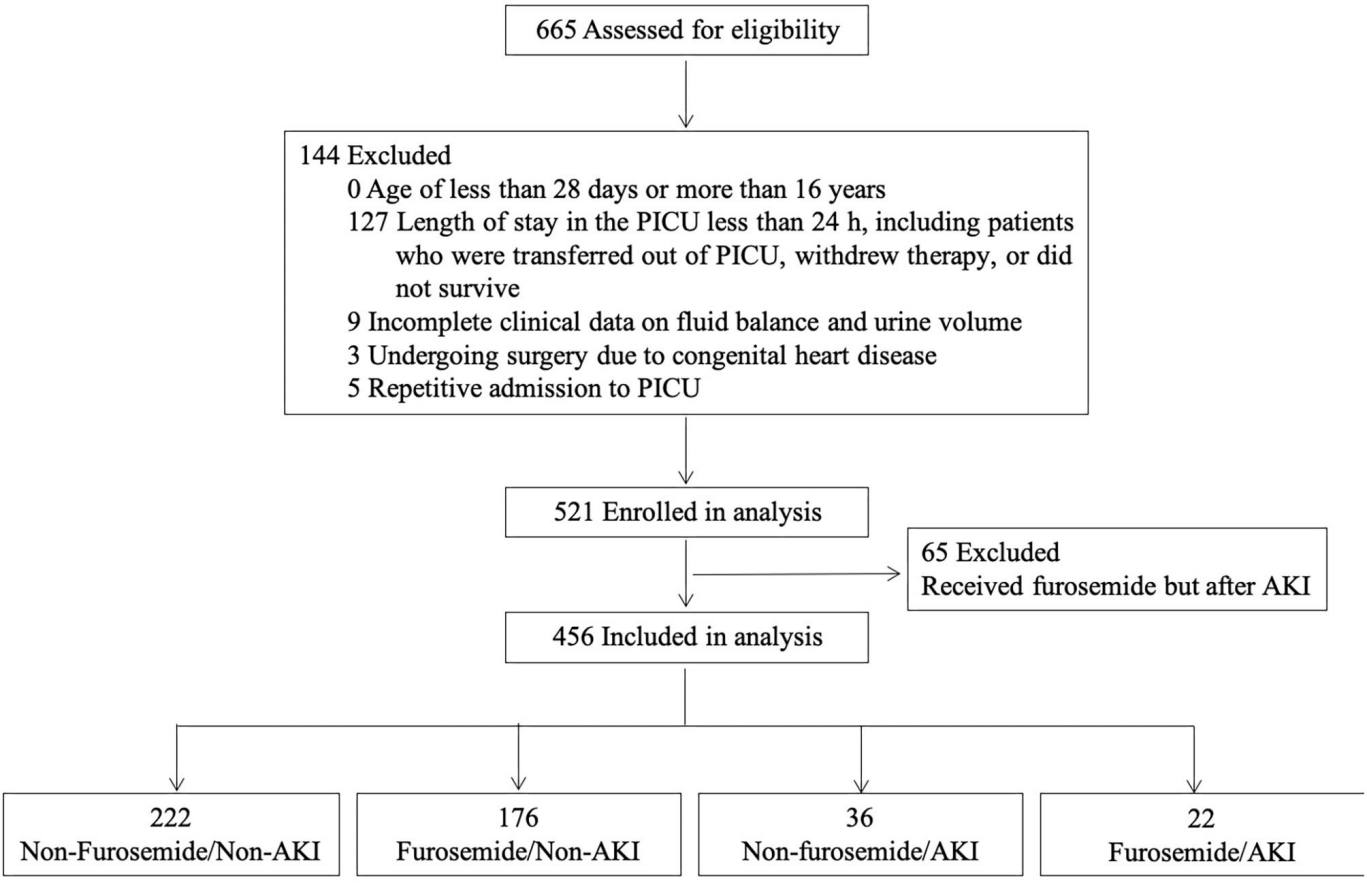
Study flow diagram.

A total of 665 patients were admitted to the PICU from January to December 2016. After the application of the exclusion criteria for the study, 456 patients were eligible for the analysis, as shown in Figure 1. The PICU mortality rate of the whole cohort was 15.4% (70/456). The median length of PICU and hospital stay was 79.0 (IQR 45.0–146.8) and 273.5 (IQR 168.0–424.5) h. The leading cause of PICU admission was respiratory diseases (34.6%), followed by neurologic diseases (16.2%), and hematological diseases (11.0%). Of the 456 patients, 371 received antibiotics, including cephalosporin, penicillin, azithromycin, vancomycin, meropenem, metronidazole, and caspofungin. No aminoglycosides were administered.

In the first week after being admitted to the PICU, 198 (43.4%) patients received at least one dose of furosemide, and 80.3% of these received their first dose within the first 48 h after admission. The median cumulative furosemide dose and frequency received in the first week after admission were 1.0 (IQR 1.0–4.0) mg/kg and 2.0 (IQR 1.0–5.0) times. As regards other diuretics, of all the patients, only one was treated with spironolactone and hydrochlorothiazide in combination with furosemide. A comparison of the demographic and clinical characteristics between patients who did and did not receive furosemide is displayed in Table 1.

**Table 1:** Characteristics of patients stratified by furosemide administration.

	**Furosemide**	**Non-furosemide**	***P***
	***n* = 198**	***n* = 258**	
Age, months	13.00 [3.00–43.50]	18.00 [4.00–64.00]	0.063
**AGE GROUP**			
≤ 12 months, *n*	97 (49.0)	109 (42.2)	0.809
>12 months, *n*	101 (51.0)	149 (57.8)	0.085
Body weight, kg	10.00 [5.18–15.00]	11.00 [6.50–18.13]	0.025
Male, *n*	114 (57.6)	152 (58.9)	0.775
PRISM III score	4.00 [2.00–9.00]	3.00 [2.00–6.00]	0.006
**ADMISSION DIAGNOSIS**			
Respiratory diseases, *n*	66 (33.3)	92 (35.7)	0.621
Neurological diseases, *n*	26 (13.1)	48 (18.6)	0.126
Hematological diseases, *n*	27 (13.6)	23 (8.9)	0.131
Accident injuries, *n*	26 (13.1)	20 (7.8)	0.062
Cardiovascular diseases, *n*	15 (7.6)	9 (3.5)	0.059
Sepsis, *n*	8 (4.0)	15 (5.8)	0.518
Gastrointestinal disease, *n*	7 (3.5)	13 (5.0)	0.496
Other, *n*	23 (11.6)	38 (14.7)	0.405
Maximum FO, %	3.91 [2.59–6.24]	3.70 [1.99–5.61]	0.066
Maximum FO ≥5%, *n*	74 (37.4)	76 (29.5)	0.087
Mean FO, %	1.10 [−0.33 to 2.61]	2.00 [0.54–3.70]	<0.001^*^
Mean FO ≥5%, *n*	8 (4.0)	37 (14.3)	<0.001^*^
oliguria, *n*	21 (10.6)	18 (7.0)	0.180
AKI, *n*	22 (11.1)	36 (14.0)	0.397
AKI stage 1, *n*	14 (7.1)	19 (7.4)	0.267
AKI stage 2, *n*	4 (2.0)	14 (5.4)	
AKI stage 3, *n*	4 (2.0)	3 (1.2)	
MODS, *n*	39 (19.7)	12 (4.7)	<0.001^*^
Shock/DIC, *n*	28 (14.1)	11 (4.3)	0.001^*^
ALI, *n*	22 (11.1)	6 (2.3)	<0.001^*^
Sepsis^a^, *n*	31 (15.7)	30 (11.6)	0.215
MV, *n*	98 (49.5)	39 (15.1)	<0.001^*^
Duration of MV, h	118.75 [55.88–213.25]	87.00 [31.15–160.00]	0.028
RRT, *n*	10 (5.1)	3 (1.2)	0.020^*^
Mannitol	61 (30.8)	56 (21.7)	0.031^*^
Vasopressor, *n*	27 (13.6)	29 (11.2)	0.473
Steroids, *n*	109 (55.1)	142 (55.0)	0.998
Antibiotics, *n*	177 (89.4)	194 (75.2)	<0.001^*^
Vancomycin, *n*	21 (10.6)	18 (7.0)	0.180
PICU LOS, h	135.5 [68.0–208.3]	61.5 [39.8–95.3]	<0.001^*^
Hospital LOS, h	349.0 [191.0–504.0]	234.5 [159.5–357.0]	<0.001^*^
Mortality, *n*	43 (21.7)	27 (10.5)	0.002^*^

*Values are median [interquartile range]. Numbers in parentheses denote percentages*.

*AKI, acute kidney injury; ALI, acute lung injury; DIC, disseminated intravascular coagulation; FO, fluid overload; LOS, length of stay; MODS, multiple organ dysfunction syndrome; MV, mechanical ventilation; PICU, pediatric intensive care unit; PRISM III, pediatric risk of mortality III; RRT, renal replacement therapy*.

* *P < 0.05, after adjustment for body weight and illness severity using analysis of covariance (ANCOVA)*.

a *Diagnosed during PICU stay*.

Of the 456 patients, 58 (12.7%) developed AKI during the first week after PICU admission. Thirty-three patients fulfilled the KDIGO criteria stage 1: 11 on the first, 9 on the second, 4 on the third, 7 on the fourth, 1 on the sixth, and 1 on the seventh days after PICU admission. Eighteen patients fulfilled the KDIGO criteria stage 2: 11 on the first, 2 on the second, 2 on the third, 1 on the fourth, 1 on the sixth, and 1 on the seventh days. Seven patients fulfilled the KDIGO criteria stage 3: 3 on the first, 1 on the fourth, and 3 on the seventh days. Characteristics of patients stratified by AKI and furosemide administration are shown in Supplementary Table 1. In addition, the baseline SCr was defined as follows: among all patients, 15 had the lowest SCr level within 6 months prior to PICU admission as the baseline, 47 had the SCr level in hospital but prior to PICU admission, 321 had the first SCr on PICU admission, and 73 presented to the PICU with an elevated SCr >106.1 μmol/L and had the lowest SCr value within 2 weeks in the PICU as the baseline SCr.

### Association Between Furosemide and Fluid Overload

Of the 456 patients, 150 (32.9%) developed maximum FO defined as ≥5% of fluid accumulation during the first week after PICU admission, including 20 (4.4%) with FO ≥ 10%. However, there are only 9.9% (45 of 456) patients who developed mean FO ≥5% in the first week. Furosemide exposure occurred in 43.4% of all patients (*n* = 456) and 49.3% in those who developed FO (*n* = 150) in the first week after admission. Exposure to furosemide was not associated with maximum FO (B coefficient = 0.043, *P* = 0.222). The incidence of maximum FO ≥5% did not differ between patients who did and did not receive furosemide (74 of 198 [37.4%] vs. 76 of 258 [29.5%], *P* = 0.087). In contrast, exposure to furosemide was associated with mean FO (B coefficient = −0.150, *P* = 0.001). The mean daily fluid accumulation was significantly lower in patients who received furosemide than in those who did not (1.10 [−0.33 to 2.61%] vs. 2.00 [0.54–3.70%], *P* < 0.001). In addition, patients who received furosemide were less likely to develop mean FO ≥5% (8 of 198 [4.0%] vs. 37 of 258 [14.3%], *P* < 0.001).

To determine whether furosemide administration was independently associated with mean FO, variables in Table 1 were analyzed by the univariate linear regression analysis, and those with a *P* < 0.05 were considered as confounding factors and entered into the stepwise multivariate analysis after excluding multicollinear variables. As shown in Table 2, the final multivariate linear regression model confirmed that the use of furosemide remained associated with mean daily fluid accumulation after adjustment (B coefficient = −0.127, *P* = 0.007). VIF and tolerance values of <2 and >0.5, respectively, indicated the absence of significant multicollinearity between variables in the final model.

**Table 2:** Final multivariate linear regression model for mean fluid overload.

	**Unstandardized coefficients**		***P***	**Collinearity statistics**	
	**B**	**Coefficient (SE)**		**Tolerance**	**VIF**
Body weight, kg	−0.300	0.068	<0.001	0.978	1.023
MODS	0.239	0.090	0.008	0.801	1.249
MV	−0.248	0.057	<0.001	0.759	1.317
Use of furosemide	−0.127	0.046	0.007	0.878	1.139

*The mean fluid overload was coded as a continuous variable. Total R^2^ = 0.13. Variables in Table 1 were analyzed by the univariate linear regression analysis, and those with a P < 0.05 were entered into the stepwise multivariate linear analysis after excluding multicollinear variables. Continuous variables with skewed distribution were log-transformed*.

*MODS, multiple organ dysfunction syndrome; MV, mechanical ventilation; VIF, variance inflation factor*.

### Furosemide and Acute Kidney Injury

Although AKI occurred less frequently among patients who received furosemide, the difference in the incidence of AKI between patients who did and did not receive furosemide indicated a non-significant value (22 of 198 [11.1%] vs. 36 of 258 [14.0%], *P* = 0.397). No association was observed between furosemide use and AKI (*P* = 0.367) or AKI stage (*P* = 0.405). In addition, there was also no difference in the occurrence of AKI between patients who did and did not receive furosemide, stratified by the maximum FO (maximum FO <5%: 23 of 182 [12.6%] vs. 11 of 124 [8.9%], *P* = 0.357; maximum FO ≥5%: 13 of 76 [17.1%] vs. 11 of 74 [14.9%], *P* = 0.825) or the mean FO (mean FO <5%: 28 of 221 [12.7%] vs. 22 of 190 [11.6%], *P* = 0.764; mean FO ≥5%: 8 of 37 [21.6%] vs. 0 of 8 [0%], *P* = 0.316).

### Association Between Furosemide and Pediatric Intensive Care Unit Mortality

The mortality rate was 15.4% (70 of 456). Death occurred more frequently in critically ill children who received furosemide than in those who did not (43 of 198 [21.7%] vs. 27 of 258 [10.5%], *P* = 0.002). The univariate and multivariate logistic regression analyses were performed to determine whether the use of furosemide was independently associated with PICU mortality in critically ill children. Variables in Table 1, including age; body weight; gender; PRISM III scores; admission diagnosis; FO and AKI developed during the first week after admission; and MV, RRT, and medication exposures developed during PICU stay were entered into a univariate logistic regression analysis. Candidate variables for the multivariate logistic regression, as confounding factors, were identified based on *P* < 0.05 in the univariate analysis.

The univariate analysis identified that furosemide use, PRISM III score (OR = 1.13; 95%CI, 1.09–1.17; *P* < 0.001), the presence of mean FO ≥5% (OR = 0.87; 95%CI, 0.78–0.96; *P* = 0.007), and AKI stage (OR = 1.68; 95%CI, 1.18–2.39; *P* = 0.004) were significantly associated with PICU mortality. The OR for patients receiving furosemide having an increased risk of mortality was 2.37 (95%CI, 1.41–4.00; *P* = 0.001) in the univariate analysis.

In the multivariate model, furosemide use remained associated with PICU mortality, even after adjustment for potential confounders, including body weight, gender, illness severity assessed by the score of PRISM III, the presence of mean FO, and AKI stage (AOR = 1.95; 95%CI, 1.08–3.52; *P* = 0.026), as shown in Table 3. We further analyzed the association of furosemide with PICU mortality in critically ill children, including 456 patients who met the inclusion criteria and 65 patients excluded who received furosemide but after the occurrence of AKI (*n* = 521). As displayed in Supplementary Table 2, the association between furosemide and mortality remained significant after adjustment for potential confounders (AOR = 2.06; 95%CI, 1.18–3.59; *P* = 0.011, *n* = 521). Moreover, there was a weak correlation between the doses (*r* = 0.197, *P* < 0.001) and the frequencies (*r* = 0.183, *P* < 0.001) of furosemide and PICU mortality.

**Table 3:** Multivariate logistic regression model for PICU mortality.

	**AOR**	**95%CI**	***P***
Body weight, kg	0.97	0.94–1.00	0.065
Gender, male	0.80	0.46–1.40	0.432
PRISM III, score	1.12	1.07–1.17	<0.001
Presence of mean FO ≥5%	1.27	0.51–3.20	0.620
AKI stage	1.46	0.97–2.21	0.068
Use of Furosemide	1.95	1.08–3.52	0.026

*AKI, acute kidney injury; AOR, adjusted odds ratio; CI, confidence interval; FO, fluid overload; PRISM III, pediatric risk of mortality III*.

To take into account the age-related difference in furosemide diuretic action, we grouped the patients into two categories: ≤ 12 months (*n* = 206) and >12 months (*n* = 250). The association between furosemide and mortality was only significant in patients aged >12 months (OR = 4.33; 95%CI, 1.90–9.87; *P* < 0.001), but not in patients ≤ 12 months (OR = 1.40; 95%CI, 0.69–2.81; *P* = 0.349). Furosemide exposure remained associated with mortality in patients aged >12 after adjustment for potential confounders, including body weight, gender, illness severity assessed by the score of PRISM III, the presence of mean FO, and AKI stage (AOR = 3.69; 95%CI, 1.38–9.86; *P* = 0.009, *n* = 250).

### Association Between Furosemide and Secondary Outcomes

Patients who were exposed to furosemide had a longer length of stay of PICU (135.5 [68.0–208.3] vs. 61.5 [39.8–95.3], *P* < 0.001) and hospital (349.0 [191.0–504.0] vs. 234.5 [159.5–357.0], *P* < 0.001), when compared to patients who were not. The univariate linear regression analysis further revealed that furosemide administration was significantly associated with a longer PICU or hospital stay, and the association remained significant after adjustment for body weight, gender, illness severity, mean FO, and AKI stage using the multivariate linear regression analysis, as shown in Table 4.

**Table 4:** Association between furosemide and secondary outcomes.

	**No furosemide vs. furosemide**		***P***
	**B**	**Standard error**	
PICU LOS, h	0.292	0.032	<0.001
	0.233^a^	0.035	<0.001
Hospital LOS, h	0.145	0.033	<0.001
	0.159^a^	0.038	<0.001

*B is the unstandardized coefficient. Continuous variables with skewed distribution, including PICU and hospital length of stay (LOS), body weight, and mean fluid overload, were log-transformed for linear regression analysis*.

*AKI, acute kidney injury; PICU, pediatric intensive care unit*.

a *After adjustment for body weight, gender, illness severity, AKI stage, and mean fluid overload using multivariate linear regression analysis*.

## Discussion

Our data indicate that furosemide exposure is associated with increased risk for mortality in critically ill children, with an absolute risk augment from 10.5% among patients who did not receive furosemide to 21.7% among patients who did. It is noteworthy that there might be no association between furosemide use and AKI in critically ill children.

In this study, furosemide administration was common, regardless of the degree of FO, occurring in 43.4% of critically ill children in the first week, and as much as 80.3% (159/198) of children received the first dose of furosemide within 48 h after admission. The reason might be due to the fact that furosemide, prescribed at the discretion of the attending specialists in the fields of critical care medicine, not only was used to treat edema but also was given as a preventive intervention. The potential benefits of loop diuretics have been demonstrated, in which diuretic use augments the average hourly urine output in critically ill patients receiving vasopressors (30), and the daily fluid balance in critically ill patients with a positive fluid balance is consistently lower, when receiving loop diuretics (12). These results suggest that diuretics can be used to achieve the early fluid control in critically ill patients by reducing FO. Similarly, our results demonstrate that critically ill children who were exposed to furosemide are less likely to develop mean FO. Regardless of what degree of the maximum FO, the use of furosemide is effective in reducing the average daily fluid accumulation, even when adjusted for potential confounders in the study.

It is reasonable to investigate the association between furosemide administration and AKI, because of the potential benefits of preventing or reducing FO (12, 20, 23, 30). Experimental evidence suggests that the mechanism of furosemide is to reduce oxygen consumption, increase renal blood flow, and inhibit the active reabsorption of sodium chloride. Thereby, the renal tubules are washed by enhancing the urine output to prevent kidney tubule injury (31, 32). On the other hand, opposing evidence exists, suggesting that renal blood flow does not increase significantly after furosemide exposure actually (33, 34). Moreover, furosemide may aggravate renal obstruction by promoting the aggregation of Tamm Horsfall protein in renal tubules, leading to AKI (35, 36). It remains controversial whether AKI *per se* can be improved by furosemide or not in recent years (12, 20, 22, 30). Our study failed to demonstrate a significant relationship between furosemide exposure and AKI, although critically ill children who received furosemide experience less mean FO. Since critically ill children were not randomized in this study, we cannot exclude the possibility that no association between furosemide and AKI could be attributed to a higher baseline risk for AKI among children who were prescribed furosemide, although we had strict criteria for inclusion and excluded 65 patients who received furosemide but after the occurrence of AKI. A multicenter randomized controlled trial is necessary to confirm our findings.

Furthermore, there is a lack of evidence-based medicine in the prevention of AKI in clinical practice. Clinical studies on furosemide and prognosis during ICU stay have reached controversial or even completely opposite conclusions (15–23). For example, a multicenter observation study demonstrates that diuretic use on the day of nephrology consultation in critically ill patients with AKI is significantly associated with an increase in the risk of death (17). However, a retrospective study in non-cardiac ICU patients with a positive fluid balance and a multicenter, multinational epidemiologic study in critically ill patients with AKI or RRT indicate that diuretic use is not associated with higher mortality (12, 16). In contrast, based on a propensity score-matching analysis, loop diuretic use is associated with lower mortality in adult patients receiving vasopressor within 48 h after ICU admission (21). To our knowledge, this study is the first investigation to determine the association between furosemide administration and clinical outcomes in a population of general and heterogenous critically ill children. Since PICU patients who did and did not receive furosemide differed systematically, it is possible that the association between furosemide and mortality might be attributed to a greater risk for mortality among children who received furosemide. Nevertheless, the multivariate logistic regression analysis strengthens the fact that there truly is an association between furosemide and PICU mortality because the association remains significant after adjustment for illness severity assessed by PRISM III score. According to our study, furosemide exposure is independently associated with PICU mortality. For every critically ill child who use furosemide in the first week, the odds for PICU mortality increased by 95% after adjustment for potential confounders.

The pathophysiologic mechanisms underlying the independent association between furosemide exposure and hospital mortality in critically ill children remain to be elucidated. We speculate that furosemide exposure should have no direct effect on death, and increased activity of the renin–angiotensin–aldosterone system (RAAS) and sympathetic nervous systems might contribute to the potential mechanisms of action through which furosemide is associated with death. As we know, the RAAS has been recognized as an important inflammatory agent associated with organ failure and mortality (37). A significant increase in the RAAS activity after diuretic exposure, especially furosemide, has been demonstrated in animal and human experiments, as evidenced by significant increases in serum renin, angiotensin II, and aldosterone concentrations and serum equilibrium concentrations of angiotensin peptides (38–40). However, the activity of the RAAS is not recorded in our study. Whether these mechanisms could be relevant in the increase in mortality seen in the present study requires further evaluation. Of note, our observation of the association between furosemide and mortality was only significant in patients aged >12 months, but not in patients ≤ 12 months, suggesting that infants may differ from older children in handling furosemide. It has been demonstrated that diuretic responses may be altered secondary to age-related differences (41). The greater power of furosemide as an anti-inflammatory agent or a bronchodilator, independent of its diuretic action, in infants has been reported in previous studies (42–44). Further research is required to confirm the association between furosemide and mortality by age category and to better characterize the different age-specific actions of furosemide in the pediatric population.

Furthermore, we use the length of stay in PICU or hospital as a dependent variable to evaluate the effect of furosemide on secondary clinical outcomes. The length of stay remained significantly longer in children who were exposed to furosemide, even after adjustment for confounding factors. It could be that furosemide exposure was significantly associated with adverse clinical outcomes, such as the increased risk of mortality and the prolonged duration in PICU or hospital. These results raise the question of whether the widespread use of furosemide in critically ill children should be discouraged. However, although the associations of furosemide use and adverse outcomes remain significant after adjustment for illness severity assessed by the PRISM III score in the study, we cannot exclude the possibility that furosemide exposure, prolonged hospital stay, and higher mortality are all reflective of severity of illness that is not captured in its entirety by the PRISM III score. It may appear obvious that critically ill children requiring furosemide exposure to increase the urine output and prevent FO might be sicker than patients not receiving furosemide. Therefore, the powerful and independent association of furosemide use with mortality warrants further consideration, and a multicenter prospective randomized controlled trial is necessary to confirm our findings and further define the causal relationship in critically ill children.

Our research leaves several limitations. First, this study is a single-center retrospective analysis. We only established an association between furosemide and mortality, and it cannot lead to the implication of cause and effect. Second, the results could be subject to selection bias by excluding a furosemide exposure after AKI during the study period, which resulted in 12.5% (65/521) of critically ill child admissions being excluded from the analysis. Nevertheless, the association between furosemide exposure and mortality remained significant in critically ill children, including 456 patients who met the inclusion criteria and 65 who were excluded (*n* = 521). Third, substantial interstudy heterogeneity exists in defining baseline SCr. Many patients have no baseline SCr available, which is common in the pediatric critical care setting. The incidence of AKI may be underestimated when SCr at PICU admission is used as a baseline; therefore, the lowest SCr value within 2 weeks in the PICU was adopted as baseline for patients with elevated SCr ≥106.1 μmol/L at PICU admission, in accordance with our previous studies (9, 10). Although it has not been validated in critically ill children, the study of Pickering et al. suggests that choosing the lowest SCr value within the first week in the ICU better approximates the true baseline distribution and leads to a similar proportion of patients being diagnosed with AKI according to the RIFLE criteria, as compared with the estimation methods of back-calculating baseline SCr (45). Fourth, nephrotoxic medications are considered as the single greatest risk factor for AKI in critically ill children (6). However, we were unable to assess potential nephrotoxic medications, including diuretics given to patients during their resuscitation prior to the PICU admission. Our study included the common nephrotoxic medications during PICU hospitalization, but the small number of patients treated limits the power to analyze the interaction among nephrotoxic medications. Fifth, we were unable to evaluate the diuretic effect of furosemide by recording the change in urine volume at 6 h after administration. Not all the patients in PICU had catheterization, and the residual urine volume of bladder was not measured. In addition, there is a deviation in the urine volume by weighing the diaper and a delay in recording the time of micturition. Sixth, our study focused on PICU patients who received intermittent bolus of furosemide. Although adverse events did not differ between intermittent and continuous administration of furosemide, a previous study showed that a continuous infusion was associated with a potential increase in 24-h urine output (46). Our results cannot be applied to continuous furosemide administration. Finally, furosemide is highly bound to plasma protein (13), and the addition of albumin to furosemide therapy in hypoproteinemic patients with acute lung injury avoids worsening of FO with better maintenance of hemodynamic stability, as compared with furosemide therapy alone (47). However, this was an observational study, and we cannot trace the therapeutic effect of furosemide combined with an albumin infusion during use. A high-quality prospective research to clarify the combined diuretic effect of furosemide and albumin infusions is needed in the forthcoming period.

## Conclusions

Our study indicates that furosemide is commonly prescribed in critically ill children with or without AKI. Although there is a potential benefit of furosemide in reducing average daily fluid accumulation and critically ill children receiving furosemide are less likely to develop mean FO, furosemide exposure might be associated with increased risk for PICU mortality, but not AKI. Multicenter prospective studies are needed to focus on the association between furosemide and prognosis and to optimize the clinical outcomes in critically ill children.

## Data Availability Statement

The original contributions presented in the study are included in the article/Supplementary Materials, further inquiries can be directed to the corresponding author/s.

## Ethics Statement

Due to the retrospective nature of the study, informed consent was waived. This study was approved by the ethics committees of the Children's Hospital of Soochow University. Written informed consent from the participants' legal guardian/next of kin was not required to participate in this study in accordance with the national legislation and the institutional requirements.

## Author Contributions

XD conceived the study, conducted data collection, analyzed the data, and drafted the manuscript. JC, WL, and ZB contributed to the conception and design of the work. XL and JW participated in the design of the study and coordination. YL had primary responsibility for study design, data analysis, interpretation of data, writing of the manuscript, and final approval of the version to be published. All authors read and approved the final manuscript.

## Conflict of Interest

The authors declare that the research was conducted in the absence of any commercial or financial relationships that could be construed as a potential conflict of interest.
