# Supplementary material for: Association Between Furosemide Exposure and Clinical Outcomes in a Retrospective Cohort of Critically Ill Children
Source: Front Pediatr. 2021 Jan 25;8:589124. doi: 10.3389/fped.2020.589124 (PMC7874070; doi:10.3389/fped.2020.589124)
Supplement: Supplementary file 1 [file Data_Sheet_1.docx]

**Supplementary Table 1** Characteristics of patients stratified by AKI and furosemide administration

|  | **Non-AKI** | | **AKI** | | **P** |
| --- | --- | --- | --- | --- | --- |
|  | **Non-furosemide**  **(n=222)** | **Furosemide**  **(n=176)** | **Non-furosemide**  **(n=36)** | **Furosemide**  **(n=22)** |  |
| Age, months | 18.0 [4.0-58.5] | 12.0 [3.0-42.8] | 19.0 [5.0-111.5]^#^ | 20.0 [9.8-61.8] | 0.070 |
| Age group |  | | | |  |
| ≤12 months, n | 96 (43.2) | 89 (50.6) | 13 (36.1) | 8 (36.4) | 0.232 |
| >12 months, n | 126 (56.8) | 87 (49.4) | 23 (63.9) | 14 (63.6) |  |
| Body weight, kg | 11.0 [7.0-18.0] | 10.0 [5.0-15.0]^*^ | 13.0 [7.0-37.8]^#^ | 10.5 [9.0-18.3] | 0.031 |
| Male, n | 132 (59.5) | 101 (57.4) | 20 (55.6) | 13 (59.1) | 0.960 |
| PRISM III, scores | 3.0 [0.0-6.0] | 4.0 [2.0-8.0]^*^ | 4.5 [2.0-10.0]^*^ | 7.5 [2.0-15.5]^*#^ | <0.001 |
| **Admission Diagnosis** | | | | |  |
| Respiratory diseases, n | 78 (35.1) | 60 (34.1) | 14 (38.9) | 6 (27.3) | 0.835 |
| Neurological diseases, n | 41 (18.5) | 26 (14.8) | 7 (19.4) | 0 (0.0) | 0.131 |
| Hematological diseases, n | 20 (9.0) | 21 (11.9) | 3 (8.3) | 6 (27.3) | 0.063 |
| Accident injuries, n | 18 (8.1) | 19 (10.8) | 2 (5.6) | 7 (31.8)^*#&^ | 0.004 |
| [Cardiovascular](file:///C:/Users/HP/AppData/Local/youdao/dict/Application/8.9.3.0/resultui/html/index.html#/javascript:;) diseases | 8 (3.6) | 13 (7.4) | 1 (2.8) | 2 (9.1) | 0.271 |
| Sepsis, n | 15 (6.8) | 7 (4.0) | 0 (0.0) | 1 (4.5) | 0.296 |
| Gastrointestinal disease, n | 10 (4.5) | 7 (4.0) | 3 (8.3) | 0 (0.0) | 0.489 |
| Other, n | 32 (14.4) | 23 (13.1) | 6 (16.7) | 0 (0.0) | 0.266 |
| Max FO, % | 3.54 [2.00-5.51] | 3.89 [2.56-6.00] | 3.98 [1.87-6.46] | 5.22 [2.88-8.40]^*^ | 0.130 |
| Max FO ≥5%, n | 63 (28.4) | 63 (35.8) | 13 (36.1) | 11 (50.0)^*^ | 0.121 |
| Mean FO, % | 2.00 [0.54-3.59] | 1.12 [-0.17-2.56]^*^ | 2.27 [0.43-3.87]^#^ | 0.68 [-0.66-3.17] | 0.001 |
| Mean FO ≥5%, n | 29 (13.1) | 8 (4.5)^*^ | 8 (22.2)^#^ | 0 (0.0)^&^ | 0.001 |
| MODS, n | 9 (4.1) | 32 (18.2)^*^ | 3 (8.3) | 7 (31.8)^*&^ | <0.001 |
| Shock/DIC, n | 6 (2.7) | 20 (11.4)^*^ | 4 (11.1)^*^ | 6 (27.3)^*^ | <0.001 |
| Oliguria, n | 13 (5.9) | 15 (8.5) | 5 (13.9) | 6 (27.3)^*#^ | 0.004 |
| ALI, n | 5 (2.3) | 18 (10.2)^*^ | 1 (2.8) | 4 (18.2)^*^ | 0.001 |
| Sepsis^a^, n | 26 (11.7) | 26 (14.8) | 4 (11.1) | 5 (22.7) | 0.449 |
| MV, n | 32 (14.4) | 85 (48.3)^*^ | 7 (19.4)^#^ | 13 (59.1)^*&^ | <0.001 |
| Duration of MV, hours | 87.3 [33.6-177.6] | 116.4 [49.8-212.6] | 87.0 [25.5-93.5] | 146.0 [76.0-322.3] | 0.129 |
| RRT, n | 3 (1.4) | 9 (5.1)^*^ | 0 (0.0) | 1 (4.5) | 0.096 |
| Mannitol, n | 48 (21.6) | 55 (31.3) | 8 (22.2) | 6 (27.3) | 0.169 |
| Vasopressor, n | 23 (10.4) | 22 (12.5) | 6 (16.7) | 5 (22.7) | 0.303 |
| Steroids, n | 119 (53.6) | 97 (55.1) | 23 (63.9) | 12 (54.5) | 0.723 |
| Antibiotics, n | 168 (75.7) | 156 (88.6)^*^ | 26 (72.2)^#^ | 21 (95.5)^*&^ | 0.001 |
| Vancomycin, n | 15 (6.8) | 18 (10.2) | 3 (8.3) | 3 (13.6) | 0.517 |
| PICU LOS, hours | 59.0 [39.0-95.3] | 134.0 [68.0-205.3]^*^ | 67.5 [43.0-113.0]^#^ | 149.0 [100.8-245.8]^*&^ | <0.001 |
| Hospital LOS, hours | 238.0 [160.8-355.3] | 337.5 [188.0-487.5]^*^ | 222.0 [123.5-376.3]^#^ | 450.0 [230.3-772.8]^*&^ | <0.001 |
| Mortality, n | 19 (8.6) | 36 (20.5)^*^ | 8 (22.2)^*^ | 7 (31.8) | 0.001 |

Values are median [interquartile range]. Numbers in parentheses denote percentages.

AKI, acute kidney injury; ALI, acute lung injury; DIC, disseminated intravascular coagulation; FO, fluid overload; LOS, length of stay; Max, Maximum; MODS, multiple organ dysfunction syndrome; MV, mechanical ventilation; PICU, pediatric intensive care unit; PRISM III, pediatric risk of mortality III; RRT, renal replacement therapy.

^*^P<0.05 vs. non-AKI/non-furosemide, ^#^P<0.05 vs. non-AKI/furosemide, ^&^P<0.05 vs. AKI/non-furosemide.

^a^Diagnosed during PICU stay.

**Supplementary Table 2** Association of furosemide use with mortality in multivariate analysis (n=521)

|  | **AOR** | **95% CI** | **P** |
| --- | --- | --- | --- |
| Body weight, kg | 0.97 | 0.95-0.99 | 0.024 |
| PRISM III, score | 1.12 | 1.08-1.16 | <0.001 |
| Presence of mean FO ≥5% | 2.23 | 1.07-4.64 | 0.033 |
| AKI stage | 1.50 | 1.16-1.92 | 0.002 |
| Use of furosemide | 2.06 | 1.18-3.59 | 0.011 |

The study population include 456 patients met inclusive criteria and 65 patients received furosemide but after AKI (n=521).

AKI, acute kidney injury; AOR, Adjusted OR; CI, confidence interval; FO, fluid overload; OR, odds ratio; PRISM III, pediatric risk of mortality III.
